# G4Boost: a machine learning-based tool for quadruplex identification and stability prediction

**DOI:** 10.1186/s12859-022-04782-z

**Published:** 2022-06-18

**Authors:** H. Busra Cagirici, Hikmet Budak, Taner Z. Sen

**Affiliations:** 1 Present Address: US Department of Agriculture - Agricultural Research Service, Crop Improvement Genetics Research Unit, Western Regional Research Center, 800 Buchanan St, Albany, CA 94710 USA; 2 Montana BioAgriculture Inc., Missoula, MT USA

**Keywords:** G-quadruplex, Machine learning, Topology, Stability, Energy, Plants, Humans

## Abstract

**Background:**

G-quadruplexes (G4s), formed within guanine-rich nucleic acids, are secondary structures involved in important biological processes. Although every G4 motif has the potential to form a stable G4 structure, not every G4 motif would, and accurate energy-based methods are needed to assess their structural stability. Here, we present a decision tree-based prediction tool, G4Boost, to identify G4 motifs and predict their secondary structure folding probability and thermodynamic stability based on their sequences, nucleotide compositions, and estimated structural topologies.

**Results:**

G4Boost predicted the quadruplex folding state with an accuracy greater then 93% and an F1-score of 0.96, and the folding energy with an RMSE of 4.28 and R^2^ of 0.95 only by the means of sequence intrinsic feature. G4Boost was successfully applied and validated to predict the stability of experimentally-determined G4 structures, including for plants and humans.

**Conclusion:**

G4Boost outperformed the three machine-learning based prediction tools, DeepG4, Quadron, and G4RNA Screener, in terms of both accuracy and F1-score, and can be highly useful for G4 prediction to understand gene regulation across species including plants and humans.

**Supplementary Information:**

The online version contains supplementary material available at 10.1186/s12859-022-04782-z.

## Background

Guanine-rich nucleic acid sequences have the potential to fold into functional secondary structures known as G-quadruplex (G4) structures [1]. In a G4 structure, four guanine nucleotides organized in a coplanar manner by flexible loop regions form a square planar structure of G-quartets (G-tetrads [2]). G4 structures are characterized by the presence of at least two stacks of G-quartets that are stabilized by Hoogsteen-type hydrogen bonding as well as by the coordination of central monovalent cations to guanine nucleotides [3]. Depending on the environment, conformation of the structure, and the sequence intrinsic features, G4s exhibit diverse topologies. The presence of cations is required for the integrity of the structure with potassium ion (K+) providing optimal effect [4, 5]. G4 structures can be formed with syn or anti conformation of glycosidic bond [2], and from one, two or four separate strands (intermolecular, bimolecular or tetra molecular), with varying topologies [6, 7]. Furthermore, G4 structure stability is dependent on the number of consecutive guanines forming G-quartets [8] as well as the length and the nucleotide composition of the loop regions [9, 10].

Although distributed throughout the genome, G4 motifs are significantly located in the functional regions of chromosomes such as telomeres as well as the regulatory regions of various genes including 5′ untranslated region (UTR), the first intron and the first exon regions [8, 11]. Growing in vitro and in vivo evidence implicates G4s in important biological processes, mainly including telomerase activity [12] and regulation of replication [13–15], transcription [16–18], and translation [19–21]. Given the corpus of evidence for regulatory and functional importance of the G4s, several tools have been developed for the genome-wide screening and annotations of the putative G4 structures.

The first computational approaches were solely based on sequence compositions. These sequence-based prediction approaches have tremendously contributed to the exploration of putative G4 structures and deciphering their biological roles at the level of the whole genome or transcriptome for various organisms. The nomenclature of G4 motifs is based on the number of consecutive G bases and the loop lengths in between. Following in vitro studies defined the sequence pattern (G_3+_N_1−7_G_3+_N_1−7_G_3+_N_1–7_G_3+_) to form stable G4 structures which has been widely accepted for nearly two decades [22]. Since then, a variety of irregular G4 motifs has been investigated to include “imperfect” G4 motifs by definition [23–25] to allow greater motif flexibility, such as longer loops, bulges, or mismatches in the G4 motif (such as those identified by the pqsfinder program [26]) based on experimentally validated structures [23]. Others focused on a new scoring system for G4s based on nucleotide composition. Because it was shown that cytosine nucleotides form strong Watson–Crick base pairing with the guanine tracks and could be detrimental for the G4 structure formation [24], later models penalized the presence of cytosine nucleotides within the G4 motif (such as in the G4-Hunter program [27]). Using either one of these approaches, large, genome-wide G4 datasets have become available for several species including main crops of wheat [28] and barley [11] as well as model species like human [29], *Arabidopsis* [30] and maize [31].

These computational approaches have limitations in the sense that they can identify where G4 motifs may be located, but they cannot inform about whether these motifs can form stable G4 structures. Biological functioning of a G4 is dependent on its ability to fold into a stable structure [32]. Structural stability depends not only on G4 structural topological features, such as the number of G-quartets, lengths of loops, and discontinuities within the G-stem [22, 23], but also external features such as cation availability and concentration [4, 5], which is hard to predict, and other internal features such as nucleotide composition and distribution [24, 27] that may affect thermodynamic stability of G4s. Consequently, several computational approaches have extended their focus on the prediction of thermodynamic stability of G4s, including Mfold [33], RNAfold [34], and CentroidFold [35]. Although most of these tools were designed for RNA, they can be modified for application to DNA, at least for secondary structure prediction. A recent study, indeed, demonstrated that in most cases, the secondary structure of DNA can be reconstructed with acceptable accuracy of a greater than 0.85 by at least one of these three tools with RNAfold showing the best overall performance [36].

An experimental approach, called G4-seq, to identify G4s in a genome-wide manner in genomic DNA, has recently been applied to humans [37]. In addition, machine learning procedures, such as Quadron [38] and DeepG4 [39], have been developed to predict G4-formation based on experimental human G4-seq datasets.

We developed a new machine learning-based prediction model, G4Boost, to define and annotate putative topological structures of G4s and predict structural stability based on secondary structure folding energy. Whilst previous tools only define G4s based on human experimental data and require the flanking regions from the G4 motif or define G4s solely on motif screening approaches, G4Boost incorporates sequence intrinsic features alone to predict the sequence topological features, quadruplex folding probability, and the secondary structure folding energy. By five-fold cross-validation approach, we evaluated G4Boost using different feature sets and across various machine learning algorithms and parameters. The final model was optimized using an extreme gradient boosting algorithm for both for the classification of folded and unfolded G4s and for the prediction of folding energy through a regression problem. Although G4Boost was developed based on plant datasets, we tested the applicability of this model to several organisms: Case studies with experimental X-ray crystallography structural data demonstrated that this model can be efficiently applied to wheat, barley, rice, maize, Arabidopsis, and human. Last, we included the performance comparison of G4Boost on the experimental X-ray data against the latest machine-learning based G4 prediction tools: DeepG4, Quadron, and G4RNA Screener to show that G4Boost outperformed these available tools in terms of both accuracy and F1-score.

## Results

### Description of the G4Boost framework

**Fig. 1:**
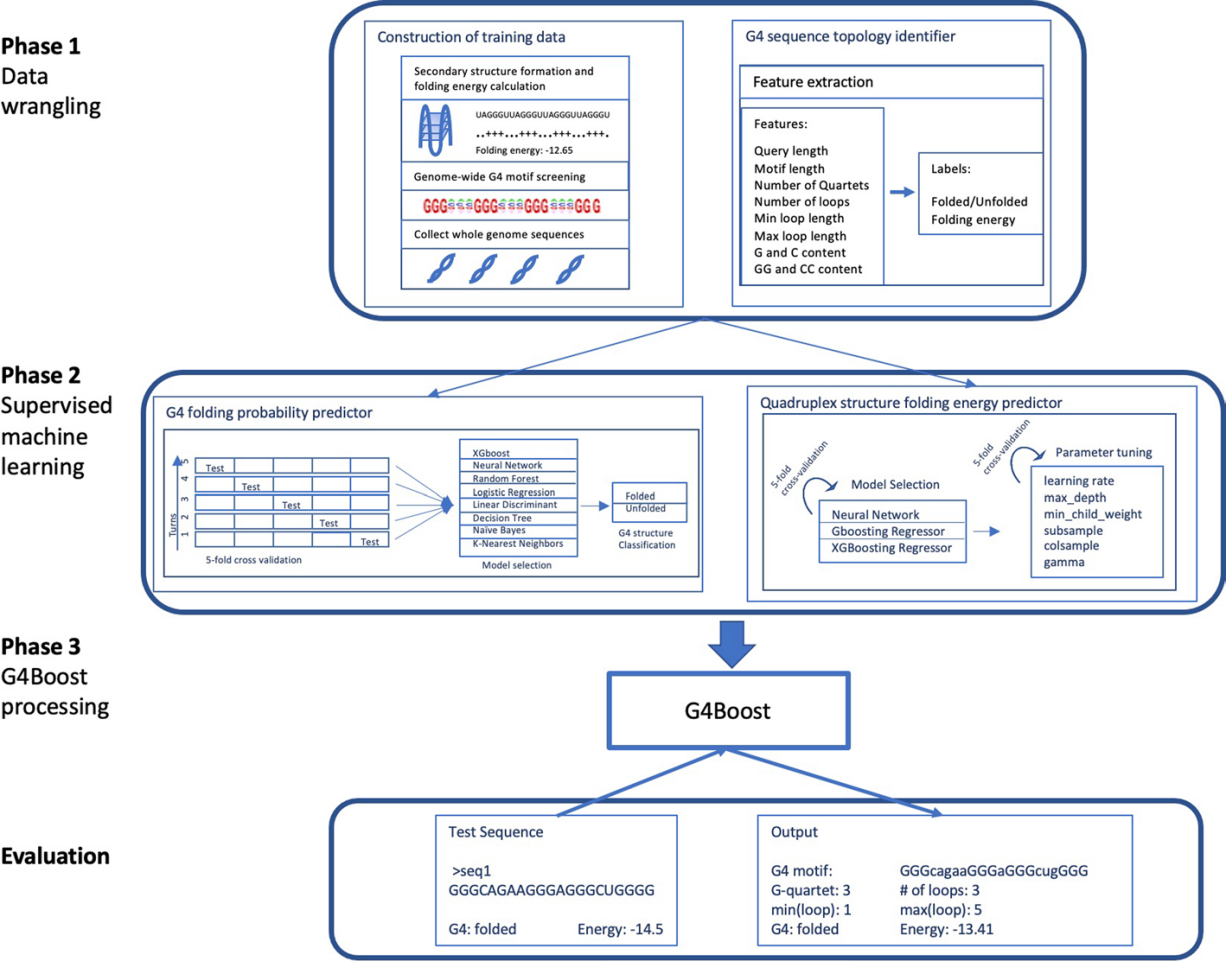
Summary of the G4Boost workflow. G4 motifs were extracted from several plant genomes and evaluated thermodynamically for folding into secondary structures to construct the training data (Phase 1). G4 motifs were described by ten features and labeled by their folding probability and energy (Phase 1). Classification models were evaluated for their prediction accuracy of the G4 structure folding (Phase 2). Regression models were evaluated and optimized for the prediction of G4 structure folding energy (Phase 2). Final prediction machinery, G4Boost, is built for the prediction of G4 structure topology, folding probability, and folding energy (Phase 3) and evaluated (Evaluation)

We describe a machine learning model for the classification and the prediction of the stability of G4 structures. G4Boost only requires an input sequence and initializes a three-layered prediction of (1) G4 structure topology, such as number of G-quartets and loop lengths, (2) G4 structure folding probability, and (3) quadruplex structure folding energy. The framework of our proposed approach is schematically depicted in Fig. 1. Phase 1 is the data wrangling step where we construct the training data and extract the G4 structure predictive features. Phase 2 is the development of supervised machine learning models for the prediction of G4 structure folding probability and folding energy. Phase 3 is the construction of final G4Boost machinery and followed by the evaluation step.

### Construction of the training data

We compiled a list of 1,720,224 nonredundant G4 motifs from a total of 35 genomes of distinct species, including Arabidopsis [40], rice [41], maize [42], wheat and barley pangenomes [43, 44], and human genome [45]. Initially G4 motifs with the most studied pattern of (G_3+_L_1-7_)_3+_G_3+_ were screened throughout these genomes on both sense and antisense directions. Although widely used, this motif has a caveat associated with it as it can give unequal number of G-bases forming G-quartets, such as “GGGtttttttGGGGGtttttttGGGtttGGG” where the second G-stem contains five guanines whereas the remaining G-stems contain three G-bases, thus two of the guanines in the second G-stem either forms bulges within the G-stem or is placed in the loop region. The first and the second loop already contain seven nucleotides and the inclusion of the two guanines from the second G-stem results in longer loops (> 7 nt) which, in fact, does not fit the initial pattern of (G_3+_L_1-7_)_3+_G_3+_. In either case of bulges and longer loops, the resulting G4s can be defined as imperfect (noncanonical) G4s [25] which were often associated with a decreased stability of the G4 structure [29]. We refined the search pattern such that G4Boost allows only equal number of consecutive G-bases in each of the G-stems and placed the additional guanines in the loop regions. This way, G4Boost does not eliminate any putative G4s, instead computes the maximum and minimum lengths of the loops and the maximum number of G-tetrads that can be formed within the input sequence.

**Fig. 2:**
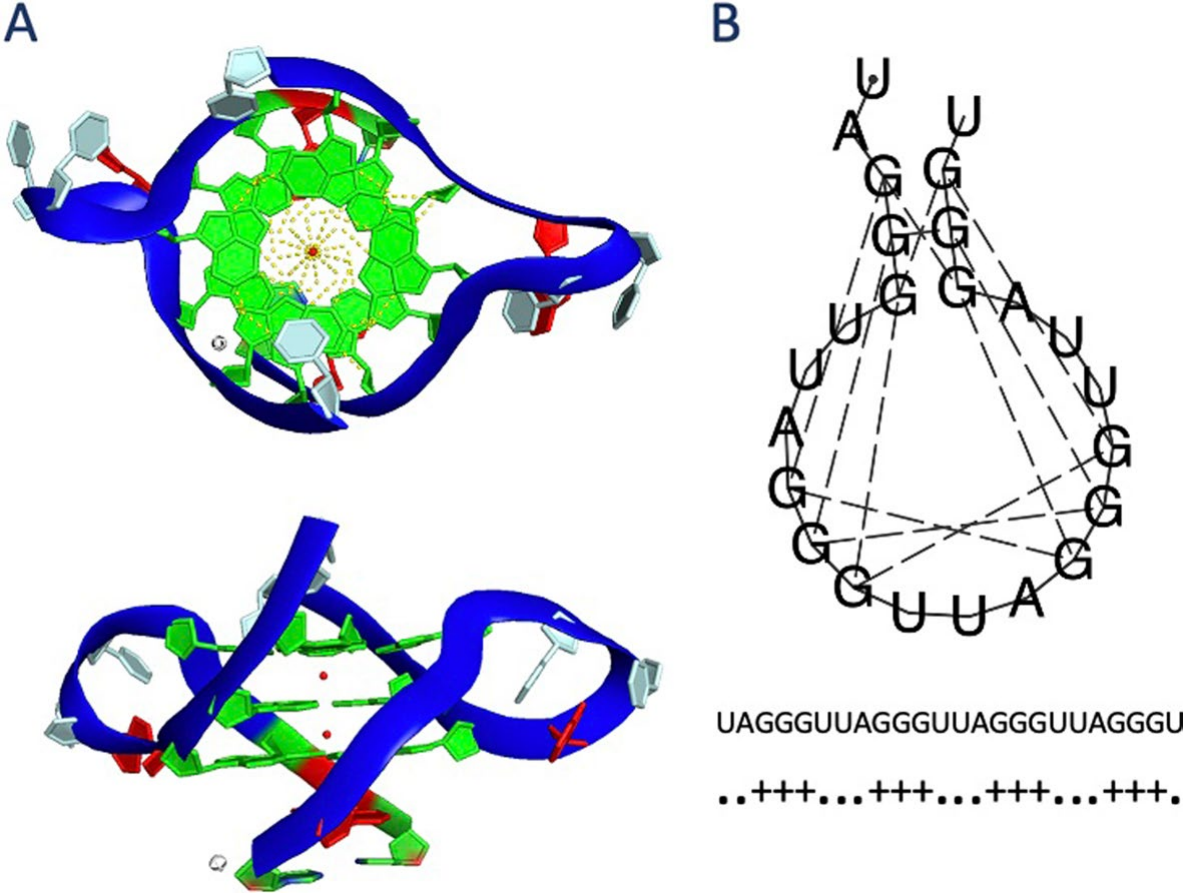
Representation of G4 structure folding. Crystal structure of a G-quadruplex (PDB ID: 3IBK [63]) from the top and the side (**A**) and its RNAfold representation (**B**) are shown. the 3D model of 3IBK is colored by its bases (guanines in green, adenines in gray, and thymines in red) and the backbone is colored blue. Two K+ ions are shown at the center. Hydrogen bonds between the K+ ion and the guanines are shown as yellow dotted lines. Bonding between the nucleotides is shown as dash lines in the RNAfold representation which is followed by the G4 sequence and the dot plus notation of the structure (**B**)

Although sequence-based prediction approaches are favorable for genome-wide motif screenings, these putative motifs are suspected to fold into quadruplex structures given the competition between the Watson–Crick and the Hoogsteen base pairing. Therefore, we further evaluate the putative G4s in terms of both sequence topology and structural stability. As a stability measure for the quadruplexes, our method uses thermodynamic energy predictions by RNAfold as structural stability label. Although designed for RNA sequences, RNAfold predicts the secondary structure of DNA sequences with high accuracy [36]. Figure 2 shows an example of G4 structure folding obtained from X-ray crystallography experiment (Fig. 2A) and predicted by RNAfold (Fig. 2B). RNAfold predicted the base pairings forming the G-quartets correctly as shown in the experimental data. The free energy of secondary structure folding was, also, calculated by the RNAfold and used as an indicator of the stability of the quadruplex.

### G4Boost identifies G4 sequence topology and locates the G4 motif in a given sequence

Supervised machine learning algorithms require training data containing labels and features. In this study, the features to calculate the folding probability and energy of the putative G4 quadruplexes were extracted based on its sequence and topology. A broad definition of G4s could lead to an infinite number of putative G4 motifs without any limits to the number of loops or the number of G-quartets “(G_3+_L_1-7_)_3+_G_3+_”. Experimental studies in humans suggested that G4s can contain longer loops around 12 nucleotide-long [46] although most of the stable G4 structures contain shorter loops (i.e., less than 7) [22, 29]. Initial definition of G4s was determined based on the most stable quadruplex topology where the number of G-quartets were limited to 3 or more. However, recent studies [47, 48] showed that although being exceptions G4 structures can be formed from less than 3 G-quartets. Therefore, we restricted our definition of G4 motifs to limit the number of loops to less than 11, G-quartets to less than 9, and loop length to 12 (expressed as “(G_1-8_L_1-12_)_3-11_G_1-8_”) to expand the G4 motifs that do not qualify under the canonical definition of G4s, i.e., “(G_3+_L_1-7_)_3+_G_3+_”_._

Our approach uses the following features: the main sequence topological features are the number of G-quartets, the number of loops, and the minimum and maximum lengths of loops; sequence intrinsic features are the lengths of query sequence and the putative G4 motif in the query sequence, in addition to the 20 features based on sequence intrinsic features, including monomer and dimer frequencies of the four nucleotides. For a total of 26 features, we assessed the contribution of each feature to the prediction models and selected the top-ranking features together with the top-ranking prediction models after 5-fold cross validations. These following 10 features were selected as top-ranking for the extreme gradient boosting algorithm: the number of G-quartets, the number of loops, the maximum loop length, the minimum loop length, the length of the query sequence, the length of the G4 motif, the composition of G and C bases, and the composition of GG and CC dimers. Ranking of all features was provided in the Additional file 4: Table S1.

### G4Boost predicts the G4 structure folding probability

We labeled the putative G4 motifs as either folded or unfolded G4s based on the thermodynamic calculations by RNAfold. RNAfold calculates the free energy for the alternative folding patterns based on base-pairing probabilities and backtracing of suboptimal structures, and returns the folding state with the lowest energy [34]. Our results showed that some of the motifs, even the canonical perfect G4 motifs, do not fold into quadruplex structures. Instead, they form stem-loop hairpin structures. Even though those sequences might yield a low folding energy, indicating higher stability, those motifs were defined as unfolded G4s in this study. We therefore developed G4Boost to predict the G4 structure folding probability of the given sequence (Fig. 2).

A reliable way to assess a machine-learning model’s performance is to train a model and test on a separate dataset [49]. In order to limit the data loss during separation of the dataset, cross-validation approaches separate a portion of the data for training and test the model on the spared data (as shown as part of Phase 2 in Fig. 1). A five-fold cross-validation approach spares 20% of the data as test and trains the model using the remaining 80% of the data. In each turn, different parts of the data are used in the test and train and the overall performance is assessed by the average scores in each turn.

A five-fold cross-validation based assessment was applied to evaluate the prediction performance of the G4 structure folding state for eight classifiers including Random Forest, Classification and Regression Decision Trees (CART), Neural Network (NeuralNet), Logistic Regression (LR), Linear Discriminant Analysis (LDA), k-Nearest Neighbors (kNN), Naïve Bayes (NBayes), and Extreme Gradient Boosting (XGBoost) algorithms. Table 1 shows 5-fold cross-validation performance metrics such as accuracy, F1-score, precision, recall, and area under the receiver operating characteristic (AUROC) using the top 10 features. All the algorithms, except NBayes, predicted the G4 structure folding with > 90% accuracy. As the features are not completely independent and scaled, NBayes provided low prediction scores (accuracy of 79%).

**Table 1:** Performance comparison of classifiers

	Accuracy		F1-score		Precision		Recall		AUROC	
	Average	SD	Average	SD	Average	SD	Average	SD	Average	SD
XGBoost	0.938	0.002	0.964	0.001	0.959	0.006	0.969	0.004	0.976	0.002
NeuralNet	0.934	0.002	0.962	0.001	0.964	0.009	0.959	0.011	0.974	0.002
RandomForest	0.933	0.003	0.961	0.002	0.956	0.006	0.966	0.003	0.963	0.005
KNN	0.928	0.003	0.958	0.002	0.955	0.007	0.961	0.005	0.937	0.010
CART	0.928	0.005	0.958	0.003	0.958	0.006	0.958	0.005	0.919	0.016
LR	0.927	0.003	0.958	0.002	0.949	0.007	0.968	0.005	0.966	0.003
LDA	0.907	0.005	0.947	0.003	0.932	0.004	0.962	0.004	0.941	0.005
NBayes	0.790	0.019	0.862	0.014	0.990	0.005	0.764	0.022	0.943	0.015

Five-fold cross-validation performance metrics of eight classifiers for the prediction of G4 structure folding probability. Average is devoted to average score and std is to standard deviation for the five-fold cross-validation sets. AUROC is the area under the receiver operating characteristic curve

Generally, accuracy is the most common metric used to evaluate a trained model; however, in this study, precision and recall are equally important since the training data is unbalanced. The trained machine learning model is expected to demonstrate balanced precision and recall performance. A good measure to evaluate the balance between the precision and recall metrics is F1-score. F1-score is usually preferred over accuracy to evaluate the prediction power of a machine learning model on an unbalanced data. Therefore, the performance of the classification was further evaluated based on statistical metrics: precision, recall, and F1-score (Table 1). Our results showed that XGBoost performs best on the training data for the classification of the folded and unfolded G4s in terms of accuracy, F1-score, recall, and AUROC (Table 1). The Neural Network classifier showed slightly better performance in the precision; however, the low recall metrics resulted in an overall F1-score comparable to XGBoost (Table 1). The G4 sequences together with the associated features that are better classified with Neural Networks than XGBoost are provided in Additional file 5: Table S2. Additional performance assessments were performed through plotting the Receiver Operating Characteristic (ROC) curve (Fig. 4) for visualization and calculating the respective Area Under the Curve (AUC) score from the ROC curve [50]. Overall, the default XGBoost classifier outperformed all the remaining algorithms for the classification of G4 folding with 93.8% accuracy, 0.976 AUROC score, and 0.964 F1-score (Table 1).

**Fig. 3:**
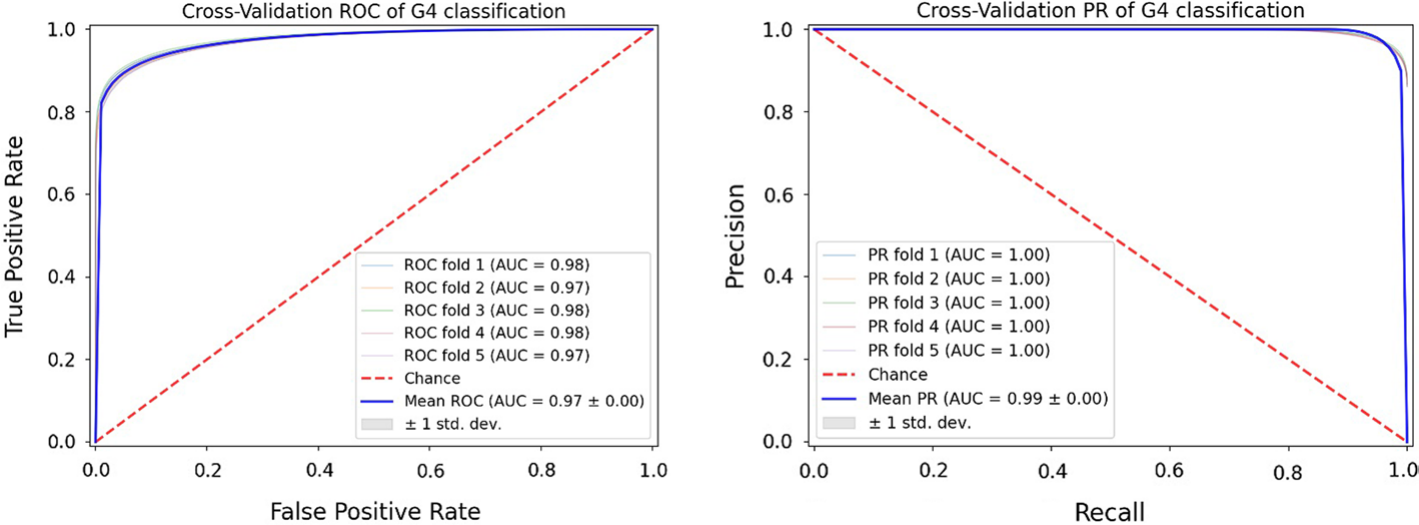
The ROC and PR curves for classification performance. The Receiver Operating Characteristic (ROC) (left) and the Precision/Recall (PR) (right) curves for the five-fold cross validation are shown. Each fold colored separately where the mean scores are colored blue, and the random probability is shown as red dash lines

We selected the extreme gradient boosting algorithm as our classifier to predict folding probability of the G4 motifs. The tree-based gradient boosting algorithm is a fast and powerful technique on a large spectrum of prediction problems, and XGBoost is among the top-performing algorithms preferred by the best machine learning competitors [51]. XGBoost algorithms are rather flexible with a set of parameters to be optimized for the best functioning. We optimized the parameters for the XGBoost classification using a 5-fold cross validation approach. Figure 3 shows the improved performance metrics after parameter optimization in the XGBoost classifier as the ROC and Precision/Recall (PR) curves.

### Feature distributions among the folded and unfolded G4s

**Fig. 4:**
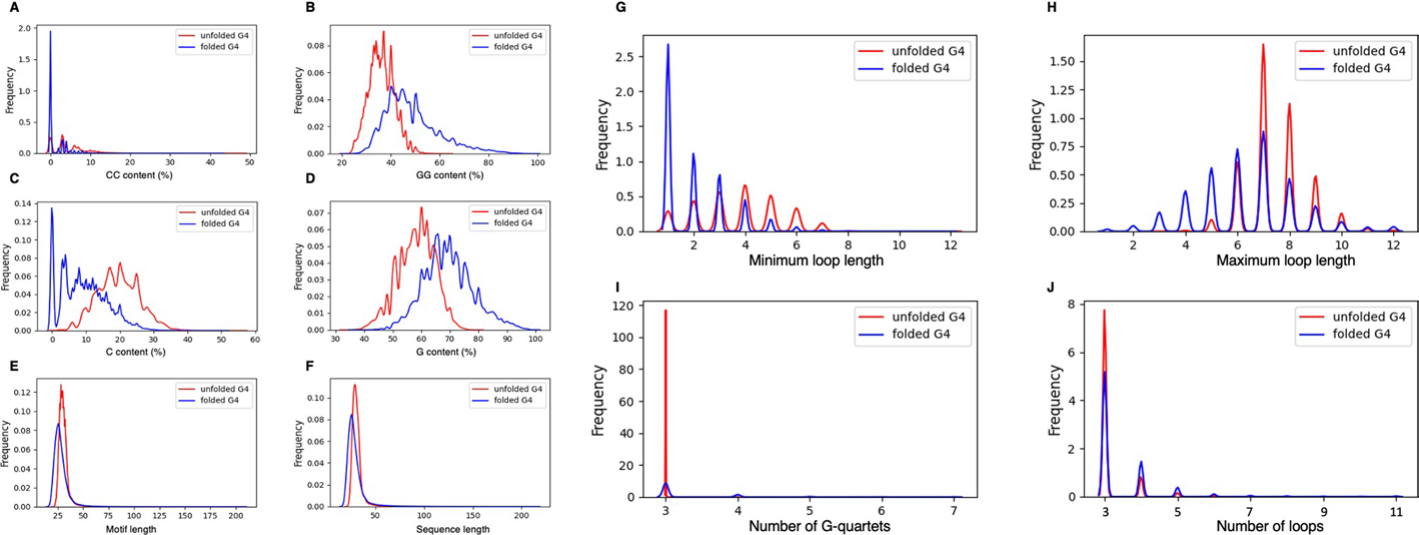
The frequency distribution of the selected features to build the prediction model for folded (blue) and unfolded (red) G4s. The features include **A** CC and **B** GG dinucleotide contents, **C** C and **D** G nucleotide contents, **E** motif length, **F** input sequence length, **G** minimum loop length, **H** maximum loop length, **I** the number of G-quartets, and **J** the number of loops

We investigated the distribution of the features and the contribution of each feature to the folding state of the G4s. Our results showed that unfolded G4 motifs in general follow the canonical definition of G4s (G_3_L_1-7_) which are almost always composed of 3 G-quartets separated by loops with a maximum length of either 7 or 8 (Fig. 4). We evaluated the training data for the refined definition of G4s and reported the location of the G4 motif within the query sequence.

Formation of stable G4 structure has been associated with the nucleotide composition of the candidate motif. In particular, cytosine nucleotides within the close proximity to the guanines in the G4 motifs could interfere with the quadruplex folding by forming strong bonds with the guanines in the G-quartets [24]. Indeed, our results showed that dimer and monomer contents of both cytosine and guanine nucleotides have high separation power for the folded and unfolded G4 motifs (Fig. 4). G and GG contents were higher in the folded G4 whereas C and CC contents were higher in the unfolded G4s (Fig. 4), suggesting that the presence of cytosines around the G4 motif could interfere with the stable formation of G4 structures.

### G4Boost predicts the G4 structure folding energy

The third layer of G4Boost predicts the G4 structure folding energy based on the thermodynamic measurements of the RNAfold. Similar to the classification of G4 structure folding, we initially compared different regression algorithms at their default settings and subsequently fine-tuned the parameters for the best-performing algorithm. Neural Network, gradient boosting, and extreme gradient boosting regression models were compared based on root mean square root (RMSE) and R-square (R2) scores as well as the explained variance and mean absolute error scores. Table 2 shows the overall performance metrics for the regression models to predict quadruplex folding energy on a five-fold cross validation set. Overall, the XGBoost model resulted in the highest scores in all the performance metrics.

**Table 2:** Performance comparison of regression models

	Variance		R2		RMSE		Absolute error	
	Average	SD	Average	SD	Average	SD	Average	SD
XGBoost	0.937	0.019	0.937	0.019	4.449	0.132	2.751	0.236
NeuralNet	0.933	0.021	0.933	0.021	4.567	0.222	2.931	0.232
GBoost	0.915	0.024	0.915	0.024	5.170	0.186	3.321	0.261

Five-fold cross-validation performance metrics of three regression models for the prediction of G4 structure folding energy are shown. Average is devoted to average score and std is to standard deviation for the five-fold cross-validation sets

**Fig. 5:**
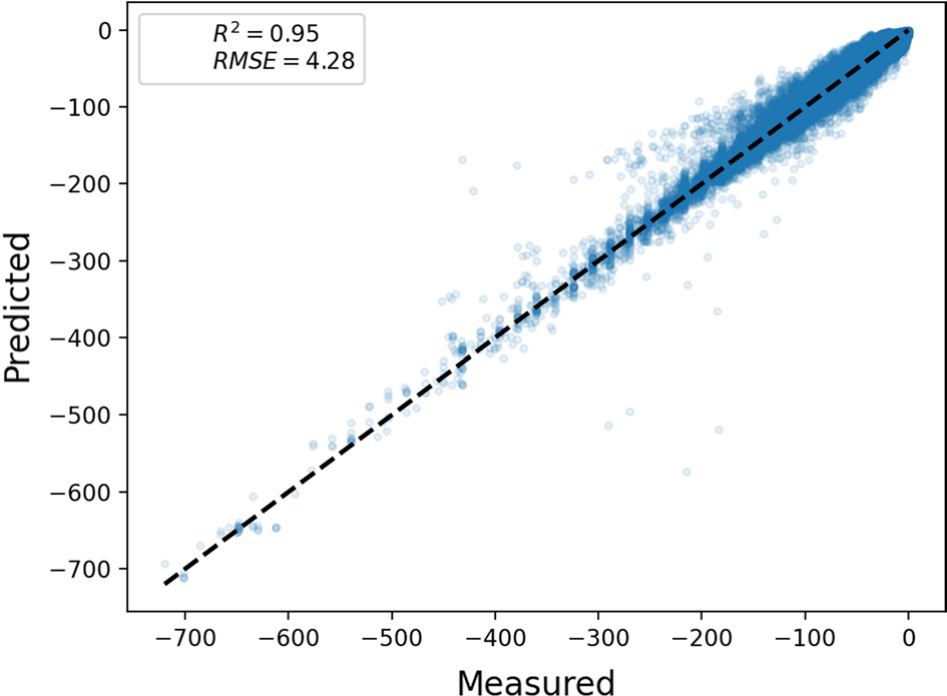
The performance evaluation of the G4 folding energy. Cross-validated predictions for each data were compared against the measured energy from RNAfold

While the baseline accuracy was high, parameter fine tuning improved the overall performance of the XGBoost regression model where the R2 score increased to 0.95 and RMSE score decreased to 4.28 (Fig. 5). Then, we assessed the features and interactions behind the model mechanics. Interrogation of the model architecture through ranking of the features by their importance revealed an assigned score for individual variables (Additional file 1: Fig. S1). We measured the feature importance in terms of weight and gain where gain is defined as the number of times a feature is visited for splitting a decision tree and the weight is defined as the empirical squared improvement as a result of this split [52]. Additional file 1: Fig. S1 shows the ranking of the features where the lengths and GG content were among the most selective features in terms of tree construction (gain) and the information gained (weight). Interestingly, the least information gained from the number of G-quartets as shown in the weight graph, although these features contribute greatly to the construction of the decision tree as shown in the gain graph (Additional file 1: Fig. S1).

Decision trees generated by XGBoost regression model include direction and reveal hierarchical dependencies of the decisions. The decision trees revealed that the first decisions were based on the number of G-quartets (Additional file 1: Fig. S2). Other decisions were independent of the context where each feature interacts with one another. Furthermore, we evaluated the training dynamics of this decision model using learning curves where we observed the correlation between the training and the cross-validation scores with a varying number of training samples. As the number of training samples increases, the training and the cross-validation scores converge together, indicating that the sample size is sufficient to construct this prediction model (Additional file 3: Fig. S3).

### Performance evaluation on the X-ray crystal structures

The prediction performance of fully trained G4Boost was evaluated on the G4 sequences collected from X-ray crystallography experiments. The Protein Data Bank [53] (PDB) website (www.rcsb.org) stores 3D shapes of proteins, nucleic acids, and complex assemblies from X-ray diffraction, NMR, and electron microscopy experiments. We collected the quadruplex data from X-ray crystallography experiments for DNA and RNA molecules only, resulting in the 238 entries, where more than half is composed of redundant sequences. Cleaning the redundancy resulted in the 78 unique G4 structures with known 3D X-ray images. Some of the quadruplexes are initially complex structures where our manual curation showed that six of the entries (1K2L, 3UKG, 6TZS, 1QYK, 6TQI, and 4GFB) are not in fact G-quadruplex structures and might be as well classified as quadruplex-like structures due to lack of bonding to form a G-quartet. This leaves the final test data with the 72 positives and 6 negatives for the folding of G4s.

As the first layer of analysis, G4Boost predicts the quadruplex topology from the sequence. Interestingly, this experimental data showed that G4s can contain G-quartets as low as 1 and with a maximum of 4 (Additional file 6: Table S3). Additionally, loop lengths showed great variance between 1 and 12 nucleotides which was, in fact, independent of our definition of G4s. As the second layer of G4 analysis, G4Boost predicts the G4 structure folding probability for the test data. G4Boost provided a 97% true positive prediction rate (70 of the 72 positives) and a 50% of true negative prediction rate (3 of the 6 negatives). Overall accuracy for the G4Boost prediction was 93.6% whereas the balance accuracy was 73.6% and the F1-score of 0.97. RNAfold, on the other hand, classified 38 of the test data as unfolded G4s, with the performance metrics: accuracy of 56.4%, balanced accuracy of 76.39%, and the F1-score of 0.69. RNAfold classified all the six unfolded G4s correctly whereas it failed to predict almost half of the folded G4s. Our results showed that although initially trained on the RNAfold predictions, G4Boost outperformed the RNAfold for the classification of the folded and unfolded G4s in terms of both accuracy and the F1-score.

As the final layer of the prediction model, G4Boost predicted the G4 structure folding energy of the test data. G4Boost predicted a folding energy of -24 on average for all the test data and the energy predictions were similar for the folded and unfolded G4s with an average energy prediction of − 24 and − 21, respectively (Additional file 6: Table S3). On the other hand, secondary structure energy predictions showed larger variance with the RNAfold when compared to cross-validation datasets. RNAfold resulted in an average energy of − 17 for the test data. Comparison of the secondary structure energy predictions between G4Boost and RNAfold yielded an RMSE score of 16.41 and an R2 score of 0.17. This discrepancy might be attributed to the inability of RNAFold to correctly classify these G4s.

### Performance evaluation against DeepG4, Quadron, and G4RNA screener

We compared the performance of G4Boost on the G4 sequences collected from X-ray crystallography experiments with the performances of the three other G4 prediction tools: DeepG4 [39], Quadron [38], and G4RNAscreener [54]. Of these tools, G4RNA screener provides an easy-to-use web server where only an input sequence is required. Quadron features a graphical user interface (GUI) as well as an executable R script than can be run provided with an input fasta file. Though Quadron requires a prior knowledge of R programming language to setup. DeepG4, on the other hand, requires experience in R scripting language to setup and run as no executable scripts is provided. In terms of G4 prediction performances, G4RNA screener and DeepG4 were able to evaluate the given sequences, whereas Quadron only evaluated the 31 of the 78 input sequences. The sequences without a favorable prediction were counted as unfolded G4s.

G4Boost outperformed DeepG4, Quadron, and G4RNA Screener in terms of accuracy, recall, and F1-score (Table 3). DeepG4 and Quadron provided the lowest true positive prediction rates of 46% and 26%, respectively. It is important to note that these two tools were initially developed to evaluate the G4s within larger regions (~ 200 bp) and evaluation of the G4 sequence itself (< 100 bp) without the knowledge of the extending regions might be limiting their performance. G4RNA Screener, on the other hand, provided a slightly better balanced-accuracy (74% vs 76%) and precision (96% vs 98%) than G4Boost as G4RNA Screener successfully classified 5 out of 6 unfolded G4s. However, due to the high false negative predictions with 22 out of 72 G4s, G4RNA Screener resulted in lower accuracy (94% vs 71%) and F1-score (0.97 vs 0.81) than the G4Boost.

**Table 3:** Performance comparison of G4Boost against other G4 prediction tools

	Accuracy	Balanced accuracy	Recall	Precision	F1-score
G4Boost	0.936	0.736	0.972	0.959	0.97
DeepG4	0.474	0.563	0.458	0.943	0.62
G4RNA Screener	0.705	0.764	0.694	0.980	0.81
Quadron	0.321	0.632	0.264	1.000	0.42

The comparison is based on the G4 sequences collected from X-ray crystallography experiments, with lengths shorter than 100 bp

## Discussion

G4s were canonically defined as at least three consecutive guanine bases separated by rather flexible loop regions up to 7 nucleotides long. Subsequent studies however showed stable structure formation with motifs even with 12-nucleotide-long, mismatches or bulges in the G-stem, or longer sequence patterns that fold into multimeric G4 structures. Adding more complexity to the canonical definition, later studies showed that cytosines within the G4 motifs might interfere with the G-quartet formation and form stable guanine-cytosine Watson–Crick pairing instead. Therefore, identification of G4 motifs that can form a stable secondary structure is a challenging task given that all G4 structures do not follow the canonical definition of G4s, and all G4 motifs with the canonical definition do not fold into stable quadruplex structures. To overcome the limitations of sequence-based predictions, in vitro evidence such as G4-seq data can be used when available to classify folding ability of putative G4 motifs via machine learning. When experimental data is not available, thermodynamic predictions can be a good alternative to predict G4 structure stability.

Here we describe a decision tree-based prediction method, named G4Boost, to predict the G4 motifs and evaluate the secondary structure folding probability and energy. G4Boost extracts G4 sequence topology features and identifies the G4 motif within a given sequence. Two key advantages of G4Boost are (i) the ability to define G4 motifs in the query sequence without considering the upstream and downstream elements if the data not available, and, more importantly, (ii) G4Boost evaluates the structural stability of the putative G4 motifs based on thermodynamic energy predictions and reports back the G4 motifs with high secondary structure folding probability. Here, we showed that G4Boost accurately predicts the quadruplex folding state (> 93% accuracy) and the folding energy (RMSE of 4.28 and R^2^ of 0.95) of the predicted sequence only by the means of sequence intrinsic feature (Figs. 3 and 5). These prediction performances were obtained with the use of XGBoost, a scalable machine learning system for tree boosting. XGBoost is powered by ensemble of multiple tree-based learners where each new tree learns from the errors of previous trees. XGBoost algorithm has shown to be superior to the most of the algorithms by being recognized as part of the winning solutions in machine learning competitions at both Kaggle and KDDCup 2015 [51].

G4Boost is not limited to humans and has been trained for a variety of species. Interspecies conservation studies showed that only less than 50% of the human quadruplexes were conserved between human and other species such as dog and mouse [55]. Furthermore, a more detailed conservation pattern of G4s across 37 species where the results showed that G4s with shorter loops are conserved whereas G4s with longer loops show diversity [56]. Since the conservation of the G4 motifs among diverse species is open to debate, covering diverse genotypes is crucial for a better understanding of the functional roles of G4s in cellular regulation.

Even though the training data was mostly composed of plant genomes, G4Boost accurately classifies human quadruplexes as shown by the experimental X-ray crystallography data for humans (Additional file 6: Table S3). For the same data, G4Boost even outperformed the available popular machine-learning based prediction tools: DeepG4, Quadron, and G4RNA Screener, in terms of both accuracy and F1-score (Table 3). G4Boost provides an accurate solution to identify and classify the G4 motifs for species and conditions for which no experimental data are available. Integrating such comprehensive data into machine learning pipelines and careful examination of the presence of G4s can lead to the identification of new pathways, and consequently may improve human health and existing plant and animal breeding programs. Therefore, we expect this three-layered G4 prediction tool will be useful in expanding our understanding of cellular and molecular control of many traits in a variety of species.

## Conclusion

Functional G4s have been associated with many important biological functions in plants and human, including but not limited to telomere maintenance [12], replication [13], a/biotic stress [11] and translation [20]. Since then large efforts have revealed candidate G4 motifs across several genomes based on sequence motif screenings [11, 28, 30, 31]. One major problem with the G4 screening approaches is that these were either solely based on sequence pattern or developed using experimental data that is available for humans. Further understanding of the G4 secondary structure folding stability is necessary because G4s have more potential to be functional when folded into stable G4 structures. Considering limited experimental data available for G4 structures, thermodynamic measurements stand as a strong candidate to evaluate secondary structure stability in plants.

We presented G4Boost to identify and annotate G4s and predict their structural stability in terms of thermodynamic energy. G4Boost does not require any additional data except the query sequence as input and is an easy-to-use python-based application. Other than direct classification, G4Boost provides a quadruplex folding probability score where different thresholds can be applied to lower or increase the confidence depending on the study.

Considering conservation variety of G4 motifs among different species, we included a wide range of species for our model. G4Boost was trained and applied to 35 genotypes and 6 species (wheat, barley, rice, maize, Arabidopsis, and human). Five-fold cross-validation experiments showed that G4Boost correctly predicts the folding state of the G4 structures with a greater than 93% accuracy, and predicts the secondary structure folding energy with high accuracy with a root-mean-square-error of 4.28 and R-squared of 0.95 for diverse species, including but not limited to plants.

Although mainly trained on plant species, G4Boost accurately classifies the experimental X-ray crystallography data for human G4s as well. An accurate prediction of G4s and their stabilities will provide a better understanding of the role of these important functional structures play in cellular and molecular regulation of traits of interest.

## Methods

All data analyses were performed in python version 3 and machine learning analysis were applied through python scikit-learn package [57].

### Construction of the G4 library

The putative G4 motifs were retrieved using a regex screening approach for the canonical G4 motif regex ('({gG}{3,}\w{1,7}){3,}{gG}{3,}') on both sense and antisense strands of the genome using regular expression operations package in python. Reverse compliments of the G4 motifs that were identified on the antisense strands were included for further analysis. We compiled a list of nonredundant G4 motifs identified from the genomes of distinct species including human (Homo sapiens—GCF_000001405.39) [45], barley (barley cultivar Morex) [58, 59], maize (Zea mays—GCF_902167145.1, B73 RefGen_v5) [42], Arabidopsis (Arabidopsis thaliana—GCF_000001735.4) [40], rice (Oryza sativa japonica—The Institute for Genomic Research (TIGR) version 7) [41], and wheat (IWGSC Chinese Spring RefSeq v1.0) [60]. To increase the coverage, we included pangenomes for barley and wheat species as well [43, 44], totaling to 35 genomes. G4 motifs identified from both sense and antisense strands of the 35 genomes were combined into one dataset and redundant sequences were eliminated.

### Thermodynamic analysis of the G4s

These G4 motifs were later subjected to secondary structure folding using RNAfold [34] with quadruplex option (-q). The G4 motifs were later classified as folded or unfolded depending on the secondary structure prediction by RNAfold. If the motif is folded into a quadruplex structure, it is classified as folded G4. If the motif is folded into other secondary structures or no secondary structure formation is observed, then it is classified as unfolded G4. Secondary structure folding energy was again calculated by RNAfold thermodynamically and was later used for the folding energy prediction.

### Feature selection

Initially we extracted 26 features based on sequence intrinsic features including initial sequence length, nucleotide content, and dinucleotide content together with the G4 sequence topology features. G4 sequence topology were defined as the motif length, number of loops connecting the runs of consecutive G bases (# of loops), the number of consecutive G bases (G-quartets), minimum number of bases in a loop, and the maximum number of bases in a loop. To remove the features contributing the least and/or for the worse performance, we applied feature selection specific to the prediction algorithms, including feature_importance functions implemented in the prediction algorithms, the recursive feature elimination, and selecting the features arbitrarily. After fivefold cross-validations, we selected the top performing combination of the features and the prediction models (Additional file 4: Table S1).

### Model construction and evaluation

Supervised prediction models were built using eight classifiers and three regressors from the python scikit-learn package. Classifiers include extreme gradient boosting, neural networks, random forests, k-nearest neighbors, logistic regression, decision trees, linear discriminant analysis, and Naïve Bayes. Regressors include extreme gradient boosting, neural network, and gradient boosting. Prediction models were trained using feature selection and parameter tuning when applicable. Hyperparameters and the codes were provided in Additional file 7: Data 1. Training accuracies were calculated by an internal five-fold cross-validation. Using the cross_validation function in the sklearn package, the prediction scores were evaluated by the mean and the standard deviations for the five cross-validation sets. These prediction scores include accuracy, balanced accuracy, precision, recall, and F1-score for classification models [61] and the root mean squared error (RMSE), the coefficient of determination (R2) score, explained variance for the regression models [62].

Additional performance assessments were performed for classification models through plotting the receiver operating characteristic (ROC) curve and the precision/recall (PR) curve for visualization. These performances were measured by calculating the area under the curve (AUC) score [50].

### X-ray crystallography data curation

Quadruplex structures were extracted from the Protein Data Bank (PDB) [53]. These X-ray determined G4 structures include intermolecular, bimolecular or tetra molecular structures. We extracted the intermolecular G4s as is, and since highly flexible loop regions do not always show up in X-ray, we modified the bimolecular and tetramolecular G4 structures by adding an arbitrary loop region between the closest strands as appropriate. The artificially added loops contained only Ts, and the number of Ts was decided based on its publication or based on the loops that were already present in the determined structure. Redundant sequences were eliminated to remove bias to obtain the final set of G4 crystal structures.

## Supplementary Information

**Additional file 1: Figure S1.** Feature importance plots of G4boost for the folding energy prediction. Gain plot on the left and the weight plot on the right represent different metrics. ‘Length’ represents the motif length identified by G4Boost and ‘seq_length’ represents the input sequence length. ‘Minlbase’ is the minimum number of bases in a loop region and ‘maxlbase’ is the maximum number of bases in a loop.

**Additional file 2: Figure S2.** XGBoost decision tree for the G4 structure folding energy. Entire decision tree predicting the folding energy of the G4s shows the readable heuristics derived from these decisions.

**Additional file 3: Figure S3.** Learning curve for XGBoost model constructed for the prediction of G4 structure folding energy. Prediction model for the quadruplex folding energy was plotted for the cross-validation sets and the full training data.

**Additional file 4: Table S1.** The importance of the features. The contributions of the initial set of 26 features were evaluated and the top-ranking features were selected.

**Additional file 5: Table S2.** The list of G4s that are better classified with Neural Networks.

**Additional file 6: Table S3.** The predictions on the test data collected from X-ray crystallography experiments.

**Additional file 7: Data 1.** G4Boost together with prebuilt prediction models and hyperparameters.

## Data Availability

All the data are available through additional files and the code is also available at https://github.com/hbusra/G4Boost.git.

## Abbreviations

AUC: Area under the curve
AUROC: Area under the receiver operating characteristic
CART: Classification and Regression Trees
G-: Guanine-
G4s: G-quadruplexes
G-tetrads: G-quartets
kNN: K-nearest neighbors
LDA: Linear discriminant analysis
LR: Logistic regression
NBayes: Naive Bayes
NeuralNet: Neural network
PDB: Protein Data Bank
PR: Precision/recall
R2: Coefficient of determination
RMSE: Root mean squared error
TIGR: The Institute for Genomic Research
UTR: Untranslated region
XGBoost: Extreme gradient boosting
