# Supplementary figures and images for: G4Boost: a machine learning-based tool for quadruplex identification and stability prediction
Source: BMC Bioinformatics. 2022 Jun 18;23:240. doi: 10.1186/s12859-022-04782-z (PMC9206279; doi:10.1186/s12859-022-04782-z)

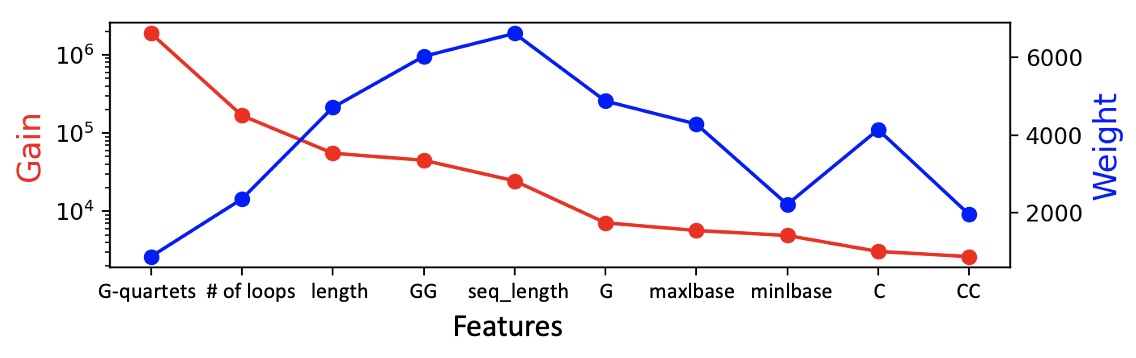

Supplement: Supplementary file 1 — Additional file 1: Figure S1. Feature importance plots of G4boost for the folding energy prediction. Gain plot on the left and the weight plot on the right represent different metrics. ‘Length’ represents the motif length identified by G4Boost and ‘seq_length’ represents the input sequence length. ‘Minlbase’ is the minimum number of bases in a loop region and ‘maxlbase’ is the maximum number of bases in a loop. [file 12859_2022_4782_MOESM1_ESM.jpg]

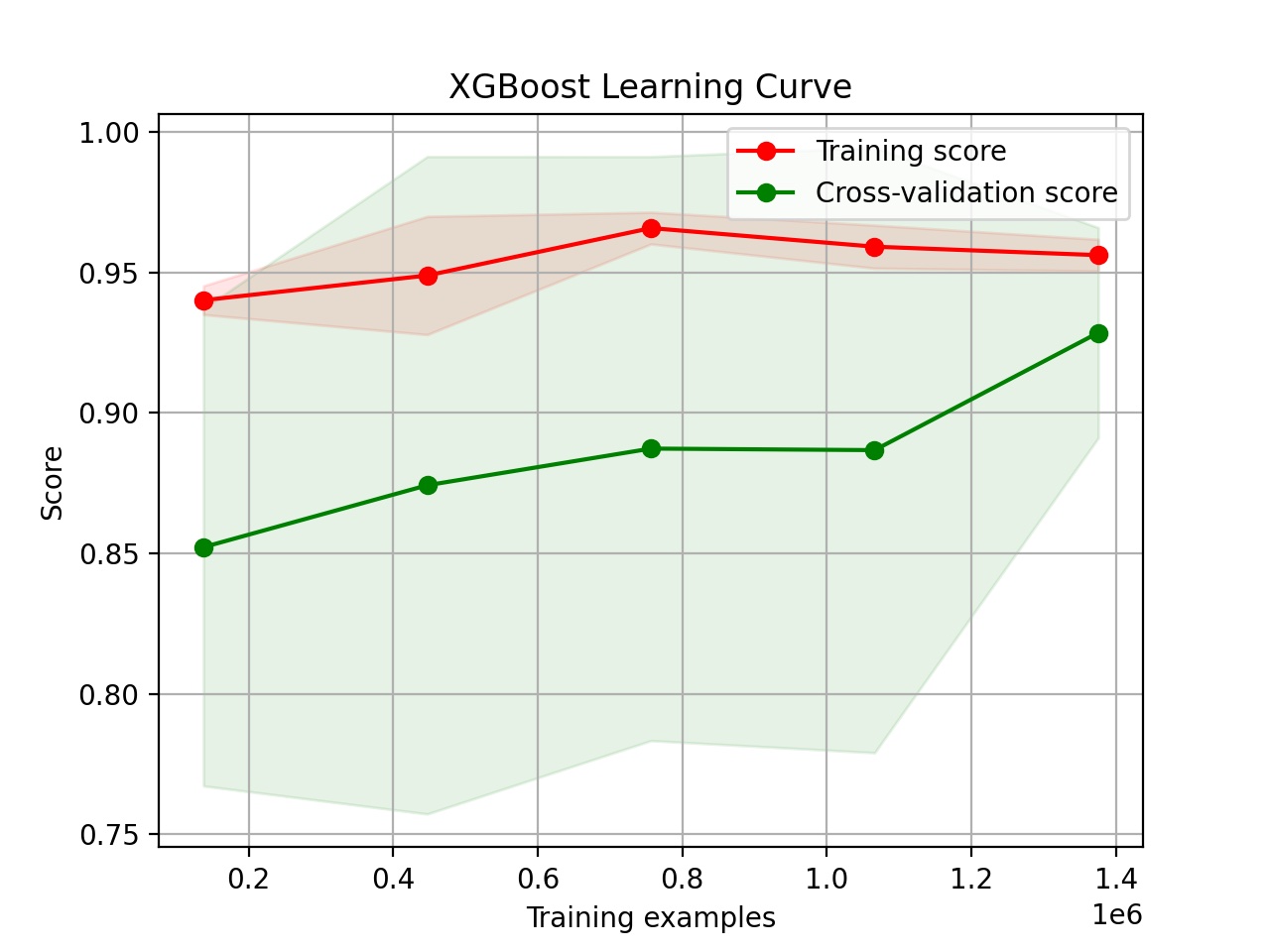

Supplement: Supplementary file 3 — Additional file 3: Figure S3. Learning curve for XGBoost model constructed for the prediction of G4 structure folding energy. Prediction model for the quadruplex folding energy was plotted for the cross-validation sets and the full training data. [file 12859_2022_4782_MOESM3_ESM.jpg]
