# Supplementary material for: G4Boost: a machine learning-based tool for quadruplex identification and stability prediction
Source: BMC Bioinformatics. 2022 Jun 18;23:240. doi: 10.1186/s12859-022-04782-z (PMC9206279; doi:10.1186/s12859-022-04782-z)

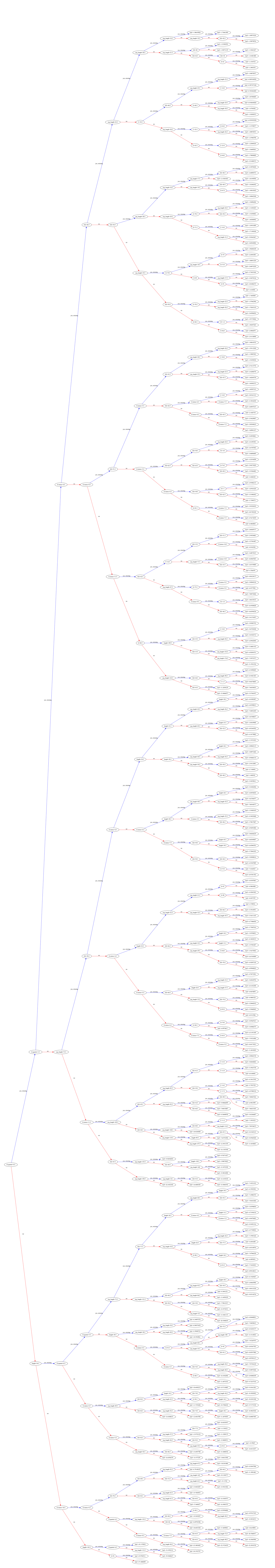

Supplement: Supplementary file 2 — Additional file 2: Figure S2. XGBoost decision tree for the G4 structure folding energy. Entire decision tree predicting the folding energy of the G4s shows the readable heuristics derived from these decisions. [file 12859_2022_4782_MOESM2_ESM.pdf]
